# SNR-Dependent Environmental Model: Application in Real-Time GNSS Landslide Monitoring

**DOI:** 10.3390/s19225017

**Published:** 2019-11-17

**Authors:** Junqiang Han, Rui Tu, Rui Zhang, Lihong Fan, Pengfei Zhang

**Affiliations:** 1 National Time Service Center, Chinese Academy of Sciences, Shu Yuan Road, Xi’an 710600, China; rockyhan@yeah.net (J.H.); zhangrui@ntsc.ac.cn (R.Z.); fanlihong321@163.com (L.F.); zhangpengfei@ntsc.ac.cn (P.Z.); 2 Key Laboratory of Time and Frequency Primary Standards, Chinese Academy of Sciences, Xi’an 710600, China; 3 College of Electronic, Electrical and Communication Engineering, Chinese Academy of Sciences, Yu Quan Road, Beijing 100049, China

**Keywords:** GNSS, SNR-dependent, environment model, landslide monitoring

## Abstract

The Global Navigation Satellite System (GNSS) is currently one of the important tools for landslide monitoring and early warning. However, the majority of GNSS devices are installed in mountainous areas and a variety of vegetation. These harsh environments lead to defective signals at high elevation angles, rendering real-time successive and reliable positioning results for monitoring difficult. In this study, an environmental model derived from signal-to-noise ratio (SNR) is proposed to enhance the precision and convergence time of positioning in harsh environments. A series of experiments are conducted on weighting and ambiguity-fixed models to evaluate performance. The results indicate that the proposed SNR-dependent environment model could lead to a significant improvement in precision and convergence time; with an obtained root mean squared result on the millimeter level, a convergence time of a few seconds, and utilization which could reach 100%, for continuous and reliable positioning results. These results indicate that the proposed SNR-dependent environment model enhances the performance of GNSS monitoring and early warning to provide continuous and reliable positioning results in real-time.

## 1. Introduction

Landslide is a major type of geological disaster across the world, especially in China. It is therefore essential to realize Global Navigation Satellite System (GNSS) high-precision landslide deformation monitoring in real-time. As one of the main technologies for landslide monitoring and early warning, real-time kinematic (RTK) techniques for GNSS based on short-baselines are widely used in geologic deformation monitoring, as they provide excellent efficiency and reliability [1,2,3,4,5]. Nevertheless, as GNSS instruments are installed in environments that contain both mountains and a variety of vegetation, which produce serious refraction and diffraction effects in terms of the GNSS satellite signals, environment severely affect the continuity and reliability of GNSS monitoring and early warning [6,7,8,9].

To solve these problems, various studies have attempted to mitigate the effects of the multipath phenomenon caused by radio-frequency reflections, using techniques that can be classified into three groups. The first group involves mitigation of multipath errors using hardware, such as a choke antenna. This is expensive, and only a part of the multipath effects are eliminated in this way [10,11]. The second group relies on date post-processing; employing methods such as a Kalman filter, an adaptive filter, Vondrak filter wavelet analysis, and a means of signal-to-noise ratio (SNR)-based weighting, all of which only effectively eliminate multipath issues by post-processing [12,13,14,15,16,17]. The last group involves mitigating multipath errors using short period repetition on the multipath, such as sidereal filter (SF) or the multipath hemispherical map (MHM) model [18,19,20,21,22,23]. Among these methods, a sidereal filter is dependent on the orbits of satellites, it is not commonly used for high-rate deformation monitoring [24]. The multipath hemispherical map model is built using an external common receiver clock, which places a limitation on the length of the monitoring baseline [20].

Although these methods might be employed to effectively eliminate the multipath errors caused by the surrounding environment, few methods meet the real-time requirements of high precision required for landslide monitoring and early warning. In 1999, a stochastic SIGMA-*ε* model was developed by Hartinger et al. while the extended weight model was proposed by Wieser in 2000 to eliminate the effects of diffraction errors. However, both methods fail in the case for which there is an unknown number of reflecting objects [25,26]. In 2006, Klostius et al. proposed that multipath errors could be significantly eliminated by an azimuth-dependent elevation mask (ADEM), which must be measured using a conventional theodolite [27]. In 2019, Han et al. suggested that the azimuth-dependent elevation mask could be derived from satellite azimuth-elevation angles, which greatly simplified the task of building the ADEM model. However, part of the work must be conducted artificially [24].

In summary, a simple and easy-to-implement model must be developed to provide high-precision continuous and reliable positioning results for real-time landslide monitoring and early warning. It is well known that the multipath phenomenon is the main error source for satellite signals. Therefore, in taking advantage of the strong correlation between satellite signal quality and the environment surrounding the antenna, an SNR-dependent environment model is proposed to eliminate the environmental influence on signals. This model could be employed to constrain and optimize the traditional GNSS algorithm in the weight model and resolve ambiguity to provide continuous and reliable resolution.

This manuscript is organized as follows: First, double-difference (DD) observation in the RTK technique, the SNR-dependent environment model, and its usage in the GNSS process are introduced. Second, the performance of the proposed environmental model is evaluated for weighting and ambiguity-fixed models. Finally, we provide our conclusions and suggestions for future work.

## 2. Models

Double-difference observation could effectively eliminate orbital, tropospheric delay, and ionospheric delay errors, and so it is widely used in RTK techniques for deformation monitoring. Furthermore, as an indicator for assessing signal quality, SNR could be employed to optimize models using RTK techniques. The following describes a basic observation model for the RTK approach, the SNR-dependent environment model, and its usage.

### 2.1. DD Measurement Model

Ignoring the effect of inter-system biases among different navigation systems, the DD observation model can be simplified as follows [28,29]:

(1) $$V_{\varphi }=\nabla \Delta \varphi -\left(\nabla \Delta \rho +\nabla \Delta M_{\varphi }+\nabla \Delta N+\nabla \Delta \varepsilon_{\varphi }\right)$$

(2) $$V_{P}=\nabla \Delta P-\left(\nabla \Delta \rho +\nabla \Delta M_{P}+\nabla \Delta \varepsilon_{P}\right)$$

where ∇Δ is the DD operator. φ and P denote the carrier phase and pseudo-range, respectively. V and ρ are the DD residuals and distance between satellites and receivers, respectively. N is the ambiguities, which can be possibly resolved as an integer using the LAMBDA algorithm [29,30,31]. M denotes the multipath effect, which is the main error source for positioning. ε is the noise, which may be ignored. After it is weighted by the elevation angle of satellites, the variance of observations $\sigma^{2}$ can be expressed as [19]:

(3) $$\sigma^{2}=a^{2}+b^{2}/{sin}^{2}\left(ele\right)$$

where ele denotes the elevation angle of the satellite. The model coefficients are a and b. The baseline vector may be resolved using the linear observation derived from Formulas (1) and (2).

### 2.2. SNR-Dependent Environment Model

A key problem related to GNSS receiver placement is that these are not always placed on suitable ground with no surrounding obstacles; and diffracted and reflected signals caused by obstacles around an antenna could be directly reflected in the SNR. This implies that the SNR value collected from different directions could be employed to build an environmental model, which could employ an appropriate algorithm to reveal the influence of the environment around the antenna. As a significant indicator of satellite observation, the SNR can be easily computed and collected from a real-time or receiver-independent exchange (RINEX) file at each frequency. If an SNR i is observed in an azimuth-elevation direction (φ,θ), then the following environmental model may be derived:

(4) $$f_{i}\left(\varphi ,\theta \right)=a_{0}+a_{1}\varphi_{i}+a_{2}\theta_{i}+a_{3}\varphi_{i}^{2}+a_{4}\varphi_{i}\theta_{i}+a_{5}\theta_{i}^{2}+\cdots +a_{m-n-1}\varphi_{i}^{k}\;+a_{m-k}\varphi_{i}^{k-1}\theta_{i}^{1}+\cdots +a_{m-1}\varphi_{i}^{1}\theta_{i}^{k-1}+a_{m-1}\theta_{i}^{k}+\varepsilon_{i},$$

where $f_{i}\left(x,y\right)$ is the SNR observation; m and k denotes the number of fitting coefficients and polynomial degree, respectively; $a_{j}$(j=0,⋯,m−1) denotes the fitting coefficients; and $\varepsilon_{i}$ is the fitting error. The matrix of Formula (1) could be expressed as follows:

(5) L=AX+ε,

where

$$A=\left[\begin{array}{cccccccccccc} 1 & \varphi_{1} & \theta_{1} & \varphi_{1}^{2} & \varphi_{1}\theta_{1} & \theta_{1}^{2} & \cdots & \varphi_{1}^{k} & \varphi_{1}^{k-1}\theta_{1}^{1} & \cdots & \varphi_{1}^{1}\theta_{1}^{k-1} & \theta_{1}^{k}\\ \vdots & \ddots & & & & & & \vdots & & & & \\ 1 & \varphi & \theta_{n} & \varphi_{n}^{2} & \varphi_{n}\theta_{n} & \theta_{n}^{2} & \cdots & \varphi_{n}^{k} & \varphi_{n}^{k-1}\theta_{n}^{1} & \cdots & \varphi_{n}^{1}\theta_{n}^{k-1} & \theta_{n}^{k} \end{array}\right]$$

$$L=\left[\begin{array}{c} f_{1}\left(\varphi ,\theta \right)\\ \vdots \\ f_{n}\left(\varphi ,\theta \right) \end{array}\right],\;\varepsilon =\left[\begin{array}{c} \varepsilon_{1}\\ \vdots \\ \varepsilon_{n} \end{array}\right]$$

$$X={\left[\begin{array}{ccc} a_{0} & \cdots & a_{m-1} \end{array}\right]}^{T}$$

*n* denotes the number of observations. With the condition of expectation E(ε)=0, the coefficient vector X can be resolved using the least square methods, producing the following expression:

(6) $$X={\left(A^{T}PA\right)}^{-1}\left(A^{T}PL\right),$$

where P is the weight matrix.

For practicality, a third-degree polynomial and the unit matrices I for ***P*** are used for convenient processing. Twenty-four hours of SNR is employed to resolve the transform coefficients. Once the transform coefficients are resolved, the environmental model that has been built for the *SNR*′, which approximate with real SNR computed from receiver, in each direction of all the satellites can be calculated using Formula (4).

### 2.3. Using the Environmental Model

As derived from SNR, the environmental model can be used as an effective indicator of quality of satellite signals. Therefore, the environmental model can be used in two parts of the positioning process for each site: One is the weighting model, the other is the partial ambiguity-fixed solution. Firstly, a virtual elevation angle, which is derived from $SNR^{\prime }$, is introduced in Formulas (7) and (8). The virtual elevation angle can be calculated as follows:

(7) $$S^{\prime }=f_{i}\left(\varphi ,\theta \right),$$

(8) $$ele^{\prime }=\frac{90.0\times \left(S^{\prime }-S_{min}\right)}{S_{max}-S_{min}},$$

where $S^{\prime }$ denotes $SNR^{\prime }$, which is derived from azimuth angle φ, elevation angle θ, and the environmental model; $ele^{\prime }$ is the virtual elevation angle that is derived from $SNR^{\prime }$; $S_{max}$ and $S_{min}$ respectively denote the maximum and minimum SNRs for the observation session of twenty-four hours.

Elevation angle is employed for weight assignment in many GNSS programs, such as GNSS at MIT (GAMIT), Bernese, and RTKLIB. However, it has been proved that the geometry-related weighting model becomes obsolete if the observations are severely affected by multipath errors, especially when the satellites signals are at a higher elevation angle (especially greater than 30°) in a harsh environment. Accordingly, an alternative weighting model derived from the virtual elevation angle is employed to eliminate the multipath error by assigning an optimal weight for each satellite in a different direction. The measurement noise of the phase range $\sigma_{L}$ can be expressed as follows:

(9) $$\sigma_{L}=\{\begin{array}{cc} F\cdot \left(31-ele^{\prime }\right)/sin\left(ele^{\prime }\right), & ele^{\prime }<E_{0}\\ 1/sin\left(ele^{\prime }\right), & ele^{\prime }\geq E_{0} \end{array}$$

where F denotes the scale factor, which depends on a specific environment, and $E_{0}$ is the threshold of the elevation angle. In this study, the two terms are set to 3.0 and 30°, respectively.

The ambiguity resolution (AR) technique may lead to greater precision in positioning by assuming that the partial phase ambiguity is an integer. As one of the most popular AR methods, LAMBDA, is commonly used in many GNSS programs. Unfortunately, not all the phase integers of satellites can always be successfully resolved, and so the partial AR (PAR) technique is chosen for reliable positioning precision. The key problem related to PAR involves the development of a principle to determine which satellites can be fixed. In this study, the virtual elevation angle is used in PAR instead of the observed elevation angle. The principle is as follows:

(10) $$m_{L}=\{\begin{array}{cc} 0, & ele^{\prime }<15{}^{\circ}\\ 1, & ele^{\prime }\geq 15{}^{\circ} \end{array}$$

where, $m_{L}$ denotes the flag to determine the satellites for which ambiguity could be fixed as an integer; $m_{L}=1$ means that it is fixed, while $m_{L}=0$ means that it is not fixed as an integer.

### 2.4. Processing Flowchart

**Figure 1:**
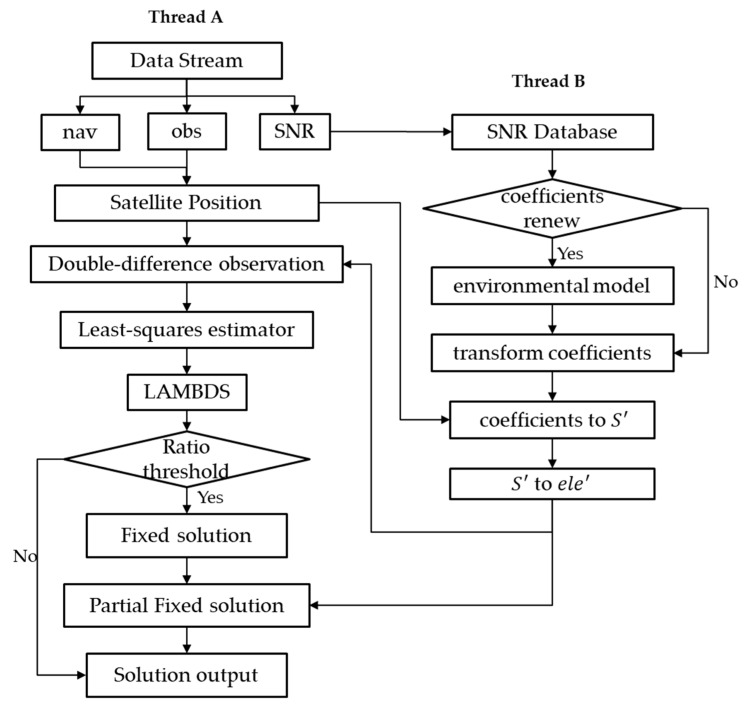
Flowchart of the signal-to-noise ratio (SNR)-dependent environmental model and its application process.

To meet the real-time requirement of monitoring, a multi-thread strategy is developed in our software package, as shown in Figure 1. One thread is used for positions, while the other is used to build and update the SNR-dependent environment transform coefficients. The main processing approach and environmental model are constructed as described below:

**Thread A:** The data streams are transformed within the wireless network to the processing center, and are then decoded to observations, ephemeris, and SNR. All the SNR are subsequently gathered into a SNR database, and the observation and ephemeris are employed to calculate the satellite position in real-time. The DD observations are then derived to estimate the float solution using a least-squares estimator. With the float solution, the fixed solution could then be resolved by LAMBDA methods. For a higher precision resolution, partial ambiguity-fixed methods are employed to promote precision.

**Thread B:** With the SNR datasets, the SNR-dependent environment model will be built using a suitable interval. The transform coefficients of the environmental model will be employed in Thread A individually in the stochastic model and partial ambiguity-fixed method, according to Section 2.3 in real-time.

## 3. Experiments and Results

A series of experiments were conducted in a mountainous region in order to provide data for testing. The datasets are then processed using the GNSS software package developed by our research group. The package achieves millimeter-scale performance for a short baseline within 10 km.

### 3.1. Data and Environmental Model

**Figure 2:**
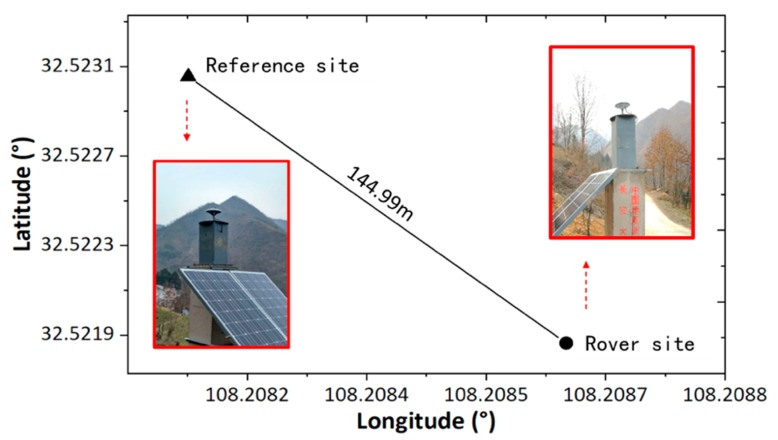
The environment around the antenna of the two sites: (left) the reference station, and (right) the rover station.

To develop the environmental model, the evaluations sites are located in the Qinba Mountain areas in Sichuan, China. Both sites are on mountainous zones and surrounded by other mountains and covered with various types of vegetation, which could lead to series multipath errors on satellite signals. The GNSS data were collected randomly on day of the year (DOY) 165 and 166 (14 June and 15 June) in 2018, respectively, for environmental model and positioning evaluation. The regions around the antennas of both the reference station and rover station are shown in Figure 2.

**Figure 3:**
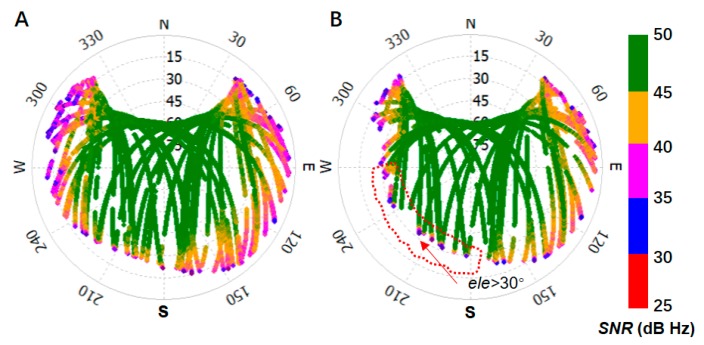
Azimuth-elevation views of SNR in azimuth-elevation angle view for: (**A**) the reference station, and (**B**) the rover station, the elevation angle about the SNR in the red dotted box is mostly greater than 30°.

As is shown in Figure 2, the reference site was mounted in a position with open sight, and the rover site was installed in a position that is surrounded by mountains. To avoid the influence of atmospheric errors, the baseline is set to a short length of 144.99 m. The instruments, including the antennas (Type: UNICORECOMM-UR380) and receivers (Type: MEASURING ANTENNA), were used for measurement and capable of tracking BDS (B1, B2), GPS (L1, L2), and GLONASS (P1, P2) signals. The sampling rate was set to one second, and the elevation cut-off angle was set to seven degrees. According to Section 2.2, twenty-four hours of SNR is collected, and the environment models for both sites were constructed through determination of the transform coefficients. The azimuth-elevation views of SNR from the reference and rover stations are shown in Figure 3.

In Figure 3, the SNR was shown by polar coordinates, where the polar angel is azimuth angle from 0° to 360° and the polar axis is elevation angle from 90° to 0°. The SNR, which had an elevation angle greater than 30° is circled by red dotted box. The value is colored from red to green as the color bar shown. From Figure 3, it can be seen in a single arc that the higher the elevation angle, the greater the SNR. Poor-quality signals were mostly concentrated at the end of the arc on each satellite, but not always at lower elevation angles for other satellites, as shown in the SNR in the red dotted box in Figure 3B. This was mostly caused by the blocking of satellite signals by physical obstacles. The boundary of the arc corresponds to the different altitudes of physical obstacles at different azimuth angles.

**Figure 4:**
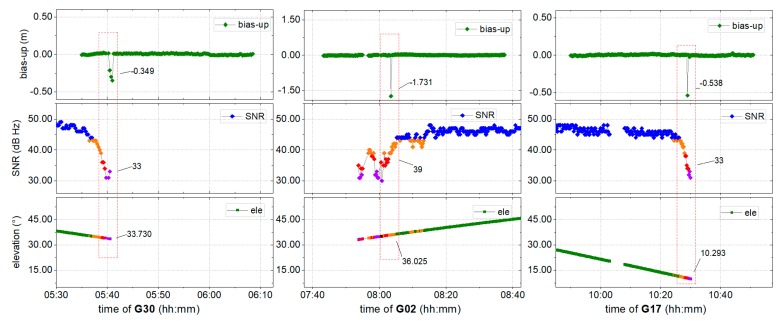
Fixed solution resolved using a traditional model. The SNR values and elevation angles of the stations with poor-quality satellite data (left) G30, (middle) G02, and (right) G17. The points certainly deviate from three correct series is shown in the red dash box, as well as the corresponding SNR and elevation angle.

To further explore the interference of unwanted signals on positioning, the fixed solution was resolved using traditional models, with the results shown in Figure 4. The corresponding SNR values and elevation angles for two satellites are also provided.

It can be seen from Figure 4 that certain abnormal points with a fixed mark deviate significantly from the correct series. From the SNR, it is clear that the majority of the signals exhibit better quality at an SNR above 40 dB Hz. Nevertheless, it also can be found that near the epoch of the jump points, there is a satellite with significantly poor quality at a higher elevation angle: The SNR of G30 was 33.0 dB Hz near 33.7°, the SNR of G02 was 39 dB Hz near 36.0°, while that of G17 was 33.0 dB Hz near 10.3°. When positioning with or without corresponding satellites, it has been shown that there is a strong correlation between abnormal results and poor-quality satellite signals.

**Figure 5:**
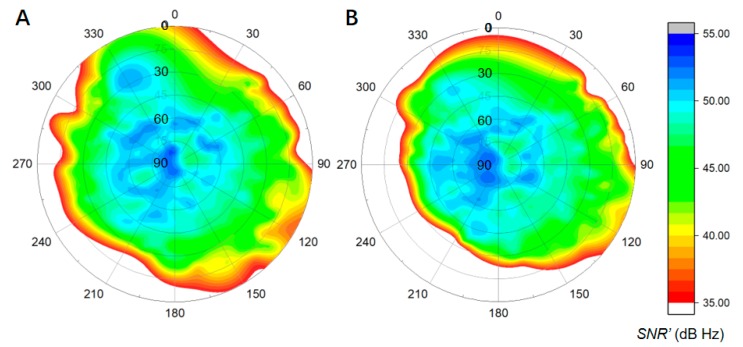
Environmental models described by *SNR*′ for: (**A**) the reference station, and (**B**) the rover station.

From Figure 3, it may be noted that many satellites exhibit poor quality near the end of the tracking arc when the receiver is installed in a mountainous position. Therefore, an environmental model that is strongly correlated with the local environment was developed according to Section 2.2. This environmental model was built for the reference and rover station sites described in Figure 2 from twenty-four hours of collected SNR data, as shown by the $SNR^{\prime }$ profiles in Figure 5.

As shown in Figure 5, the *SNR*′ observations exhibit a distribution that is coincident with the SNR data shown in Figure 3. The *SNR*′ profile of the rover station (B) exhibits a significant deviates away from the bottom left-hand corner of the view, while that of the reference station has a symmetrical form. The signals at less than 40 dB Hz are distributed around the edges of both views.

**Figure 6:**
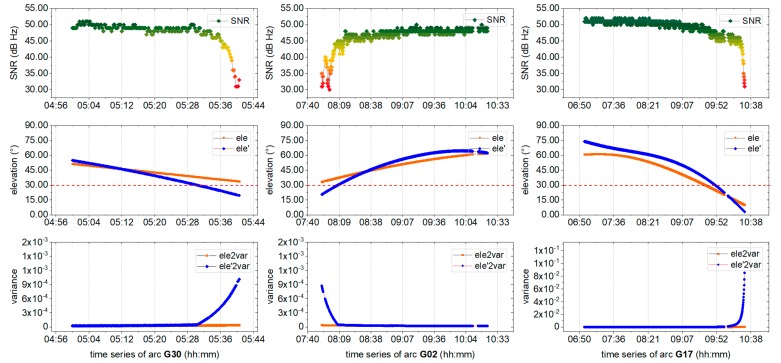
SNR, elevation angles, and variances for the G30, G02, and G17 satellites. The SNR (gradual color change from green to red) are provided for each satellite. Elevation and variance (blue), which are both derived from the environment model, are compared with the measured elevation angle and corresponding variance (orange).

To evaluate the performance of the environmental model, the SNR, elevation angles, and variances of three randomly satellites (G30, G02, and G17) that are related to bad positioning epochs are compared. The virtual ele *ele’* and the corresponding variance (*ele’2var*) were both derived from the environmental model described in Section 2.3, and are shown in Figure 6. The measured elevation angle *ele* and the corresponding variance (*ele2var*) were also provided for comparison, together with the SNR. Changes in the SNR are shown in the gradual color change from red (bad) to green (good), *ele’* and *ele’2var* are shown in blue, while *ele* and *ele2var* are shown in orange. The dashed red line marks the 30° elevation angle.

Figure 6 shows that, although the measured elevation angles of the satellites are all greater than 30°, the SNR are of a lower quality, of less than 40 dB Hz, and the variance derived from *ele* is not adjusted consistently with decreasing signal quality. Fortunately, the variance derived from the virtual elevation angle *ele’* is sensitive to signal quality, changing consistently with signal quality. It implies that the environmental model could correctly reflect signal quality at higher elevation angles, which could be employed in a positioning model for increased positioning precision.

To further evaluate the performance of the SNR-dependent environmental model in terms of positioning, a series of experiments are conducted with and without the proposed strategy. The results in the different modes are provided in terms of: (A) the float solution, (B) the ambiguity-fixed solution, and (C) partial ambiguity resolution.

### 3.2. Performance Evaluation of the Float Solution

**Figure 7:**
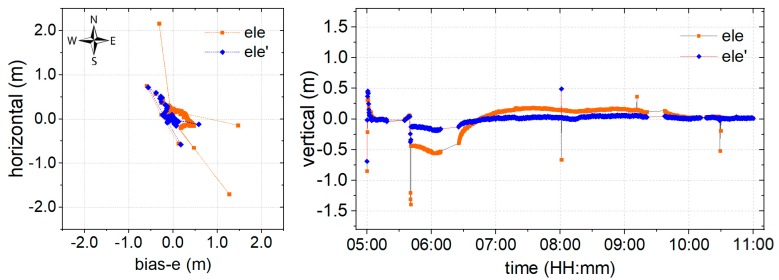
Coordinate bias of the float solution for the classical (orange) and proposed (blue) model in the (**left**) horizontal and (**right**) vertical directions.

It is well known that the float solution for positioning could provide centimeter-level monitoring precision after convergence. To evaluate the performance of the proposed environmental model in the stochastic solution, the experiment is conducted using a float solution. The data at DOY 166 is processed using the classical (orange) and proposed (blue) models, as shown in Figure 7, where deviations of the coordinate bias in the time series are illustrated in the horizontal and vertical components. The north arrow is used to indicate the specific component, and bias-e denotes the deviation of the coordinate bias in the east component.

The results of a session from time 5:00−11:00 are provided in Figure 7. The breaks in the series were caused by missing observations, as the data was collected in real-time. Certain outlying points were also noticeable. It may be suggested that these outlying points are caused by satellites with poor quality signals at a higher elevation. Compared with the results of the classical model, the proposed model shows a significant improvement both in terms of precision and convergence time. According to the statistical analysis, the root mean squared (RMS) result for the series that was obtained using the proposed model is 0.059 m, 0.061 m, and 0.067 m in East, Up, and North (EUN) components, respectively. This shows an improvement of 56.29%, 57.34%, and 66.67%, respectively, in the EUN components. The convergence time also improved of 68.39 from greater than half an hour for the classical model, to less than half an hour. Accordingly, it can be concluded that the proposed environmental model could definitely promote increased precision and reduced convergence times for the float solution.

### 3.3. Performance Evaluation of the Ambiguity-Fixed Solution

**Figure 8:**
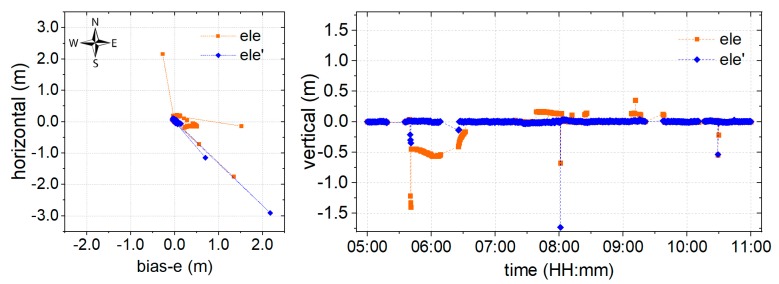
Coordinate bias of the ambiguity-fixed solution with the classical (orange) and proposed (blue) models in the (**left**) horizontal and (**right**) vertical directions.

Although the float solution could provide high monitoring precision, a long time is required for convergence of the phase ambiguity. This may not meet the demands of real-time monitoring. For this reason, the fixed solution may always be employed for positioning in real-time. In this study, the well-known LAMBDA technique [29,32] was employed for ambiguity-fixed solutions, and the threshold of the ratio is set to 3.0, as in other studies [33]. The deviations of the coordinate bias using the classical (orange) and proposed (blue) models are shown in Figure 8.

As is shown in Figure 8, the convergence times of the ambiguity-fixed solution was significantly shorter than that of the float solution. The RMS results show that the precision of the proposed model is significantly improved, by 52.95%, 18.58%, and 69.71% in EUN components, respectively. The RMS of the results obtained using the developed model in all directions reaches the centimeter-level, with 0.064 m, 0.092 m, and 0.053 m in EUN components. In addition, a significant improvement in reliability is obtained, since the results are almost fixed, with a fixed success rate of 97.8%, while the classical model only provides a fixed success rate of 77.5%. These results suggest that the proposed model provides improved precision, together with a fixed success rate.

### 3.4. Performance of Partial Ambiguity-Fixed Solution

**Figure 9:**
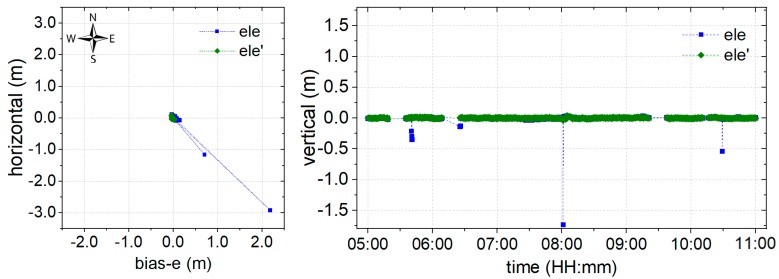
Coordinate bias of partial ambiguity-fixed solution with the classical (blue) and proposed (green) model both in the (**left**) horizontal and (**right**) vertical directions.

Partial AR is employed to improve the resolution reliability. As shown in Figure 4, the phase ambiguity in a few epochs has been marked fixed, but the positioning results were incorrect because of unreliable integer ambiguity. This was determined to mainly originate from poor-quality signals, while satellites are at a higher elevation angle. Hence, as shown in Figure 9, an alternative model (green) derived from the proposed environmental model is employed for the partial AR component, according to Section 2.3.

As is shown in Figure 9, all the outlying points have been corrected using the proposed model. Precision is improved by 89.06%, 88.04%, and 86.69% in the EUN components, and millimeter-level precision has been reached, with an RMS of 0.007 m, 0.011 m, and 0.007 m in the EUN components. The ambiguity-fixed solution success rate can reach 100%. These results indicate that the proposed environmental model could further improve the reliability of positioning using partial ambiguity resolution.

### 3.5. Discussion

The data collected from the real mountainous environment are employed to evaluate the performance of the model proposed in Section 2.2. It can be seen from Figure 3 that the satellite signal quality, as well as the range of the azimuth-elevation view in the mountainous area, are worse than in more open areas. Many satellite signals would inevitably be of a lower quality due to the influence of obstacles, especially at high elevation angles in a mountainous area. That leads to a situation in which traditional models that employ weighting by elevation angle do not work [17,24,27], and leads to a serious reduction in positioning performance, not only in terms of precision but also in convergence time, as shown in Figure 7. Models such as MHM that are proposed in the literature [20], are mostly dependent on the sidereal day, which is hardly determined in real-time.

For these reasons, an environmental model derived from SNR, which is strongly correlated to the physical environment around the GNSS antenna, is proposed to reduce the weight of poor satellite signals in positioning. The proposed model has been derived using weighting, AR, and partial AR models, and the corresponding performances are shown in Figure 7, Figure 8 and Figure 9, respectively. From Figure 6, it can be noted that the environmental model correctly reflects satellite signal quality, especially at higher elevation angles. Accordingly, the proposed model is employed to eliminate the multipath effects of the environment in a harsh area. From Figure 7, it can be seen that the proposed weighting model provides better performance of weighting to calculate a proper variance for the satellites with poor quality, especially at higher elevation angles. And, the solution RMS improvement of 56.29%, 57.34%, and 66.67% in the EUN components. Figure 8 shows that the ambiguity-fixed case could lead to a further improvement in precision, by 52.95%, 18.58%, and 69.71% in the EUN components. Although an improvement is made by ambiguity-fixed technique based on the improved float ambiguity in float case, the influence of poor quality satellites’ observation, such as G30, G02, and G17 could not eliminate exhaustively. Fortunately, the environmental model could be employed to decide which satellite ambiguity could be resolved as integer in partial ambiguity-fixed case according to Formula (10). Figure 9 indicates that the partial ambiguity-fixed case correctly excludes the satellites with poor quality, such as G30, G02, and G17, and provides further improvements in the precision, where the RMS is improved by 89.06%, 88.04%, and 86.69% in the EUN components. The data in Figure 7, Figure 8 and Figure 9 suggests that, with the proposed models that are derived from environmental factors, precision could reach the millimeter level, and the utilization of positioning results could approach 100%.

## 4. Conclusions

When a GNSS antenna is installed in a mountainous area for monitoring, the signals of most satellites are affected by obstacles around the antenna, especially at high elevation angles. That leads to reduced positioning performance in terms of precision and availability. An environmental model derived from SNR is therefore proposed to eliminate the influence of poor-quality signals. The environmental model is deployed to update a weighting model and an ambiguity-fixed model, and a series of experiments are conducted to evaluate the performance of the proposed approach. From these experiments, it can be concluded that:

(1) Weighting using the proposed environmental model could effectively lessen the influence of poor signals, and provide an improved RMS and convergence time compared to that obtained using elevation angle. However, certain abnormal epochs caused by poor signals at higher elevation angles cannot be resolved.
(2) Based on float ambiguity resolution using the proposed weighting model, the ambiguity-fixed solution is resolved. It can be shown from the results that the proposed environmental model significantly improves the success rate of the ambiguity-fixed solution, which plays an important role in providing a continuous and reliable indication of landslide monitoring and early warnings. However, abnormal epochs are not eliminated in this manner.
(3) Employing the partial AR model, which is derived from the proposed environmental model, positioning precision is further improved and even reaches the millimeter level, both in the horizontal and vertical components. Furthermore, the abnormal epochs are eliminated, which could effectively prevent false alarms during monitoring.

Although the proposed environmental model could be employed to optimize positioning for continuous and reliable landslide monitoring, the model is built using a single navigation system in our program. The development of an environmental model that employs multi-GNSS is the focus of our future work.
